# *Janthinobacterium lividum* as An Emerging Pathogenic Bacterium Affecting Rainbow Trout (*Oncorhynchus mykiss*) Fisheries in Korea

**DOI:** 10.3390/pathogens8030146

**Published:** 2019-09-11

**Authors:** Woo Taek Oh, Sib Sankar Giri, Saekil Yun, Hyoun Joong Kim, Sang Guen Kim, Sang Wha Kim, Jeong Woo Kang, Se Jin Han, Jun Kwon, Jin Woo Jun, Se Chang Park

**Affiliations:** 1 Laboratory of Aquatic Biomedicine, College of Veterinary Medicine and Research Institute for Veterinary Science, Seoul National University, Seoul 08826, Korea; mike0202@snu.ac.kr (W.T.O.); giribiotek@gmail.com (S.S.G.); arseidon@snu.ac.kr (S.Y.) hjoong@snu.ac.kr (H.J.K.); imagine0518@snu.ac.kr (S.G.K.); kasey.kim90@gmail.com (S.W.K.); kck90victory@naver.com (J.W.K.); sejin.n@snu.ac.kr (S.J.H.); kjun1002@snu.ac.kr (J.K.); 2 Department of Aquaculture, Korea National College of Agriculture and Fisheries, Jeonju 54874, Korea

**Keywords:** *Janthinobacterium lividum*, *Oncorhynchus mykiss*, Korea, pathogen

## Abstract

*Janthinobacterium* spp. are normally considered non-pathogenic, and few pathogenesis-related studies have been reported. Here, we report the first isolation of *Janthinobacterium lividum* in Korea as a pathogenic bacterium infecting rainbow trout. Mass mortality was observed at one rainbow trout hatchery, and dead fish were necropsied. Gram-negative, nonmotile, rod-shaped bacteria that grew on Cytophaga agar were isolated. A specific violet pigmentation was observed after 7 days of cultivation, and the species were characterized on the basis of the analysis of the 16S rRNA gene. Because no research has focused so far on the pathogenicity of these bacteria, our study was directed to their pathogenic role based on infection-induced histopathology. Examination of stained tissue sections revealed severe renal bacteraemia and tubule degeneration. Other tissue sections, including sections from the liver and the spleen, were relatively clear. The measured half-maximal lethal dose (LD_50_) was approximately 3 × 10^5^ colony-forming units/fish, suggesting that this bacterium may be an opportunistic pathogen in rainbow trout fisheries. Since the bacterium commonly dwells in soil and most water for rainbow trout fisheries in Korea is supplied from ground water, the bacteria may naturally flow into the aquatic environment. Therefore, recognition of any pathogenic role of *J. lividum* is important for the prevention of disease in aquaculture.

## 1. Introduction

*Janthinobacterium lividum* is a purple-pigmented rod-shaped Gram-negative bacterium of the phylum *Proteobacteria*, family *Oxalobacteraceae* [1]. It is commonly isolated from the microbiota of forest soils, Antarctic glaciers, and lake and river water [2]. Because of its distinctive violet color, the species was named on the basis of the Latin word “*janthinus*”, which means violet. The pigmentation is the result of the compound violacein, which is produced by the biochemical activity of the organism during metabolism [3]. This production of violacein is a response of the microorganism to environmental stresses and contributes to its defence against external danger [3]. Violacein is produced by bacterial strains of various genera, including *Janthinobacterium*, *Duganella*, *Collimonas*, and *Pseudoalteromonas* [4]. In addition, violacein is known to have antibacterial, antiviral, and antifungal properties, with the antifungal effects being widely exploited by amphibians to protect themselves from major pathogens such as *Batrachochytrium dendrobatidis* [5].

The antibacterial effects of violacein have been investigated in many studies of multidrug-resistant pathogens, including methicillin-resistant *Staphylococcus aureus*, vancomycin-resistant enterococcus, and fluoroquinolone-resistant *Pseudomonas aeruginosa* [6]. However, while the distinctive multiple-inhibitory effects of violacein have been explored, the pathogenicity of its bacterial source has been largely overlooked.

Clinical research studies of *J. lividum* in rainbow trout (*Oncorhynchus mykiss*) were first reported for small fish farms located in Scotland and Northern Ireland in 1992 [7]. Subsequently, there have been no reports regarding *J. lividum* as a pathogen at fisheries. There are a few reports of this bacterium being a component of the gut microbiota of marine fish, while it has been found in the gut of farmed Atlantic salmon [8]. Because *Janthinobacterium* spp. are also commonly present in river water, they are considered a basic group of fish gut microbiota, present also in mullet and menhaden [8]. However, depending on the immune status of the fish and the presence of other pathogens affecting the status of fish health, such as *Aeromonas*, *Vibrio*, and *Pseudomonas* species, *Janthinobacterium* spp. may have pathogenic roles in aquatic environments [9].

Since no research has been conducted regarding the pathogenic role and infection mechanism of *Janthinobacterium* spp., our study focused on understanding its infection process through histopathological interpretation. Moreover, this is first time reporting *Janthinobacterium* infection in Asia country aquaculture as a causative agent of mass mortality. In May 2018, one of the rainbow trout farms located in Chungbuk Province, Korea, requested an investigation of the cause of increased rainbow trout mortality on the farm. The farm cultured 600,000 rainbow trout fry that were distributed in 20 water tanks. Usually, the mortality rate was <5% but had increased to 25%. Therefore, we decided to investigate the probable cause of the increased death rate and to attempt to identify a way to treat the disease.

## 2. Materials and Methods

### 2.1. Fish Sampling

A disease outbreak occurred on a rainbow trout fish farm located in Chungbuk Province, Korea, in May 2018. The temperature and dissolved oxygen levels of the water in which the fish were raised were approximately 18 °C to 20 °C and 8 to 9 ppm, respectively. The tanks on the rainbow trout farm were separated according to the size of the stocked trout, with each tank containing an average of 3.5 tons of fish. The diseased and dead fish were collected every morning and sent daily for diagnosis for 10 days. This time frame was chosen because the greatest fish mortality occurred over this period and decreased afterward. The dead fish were collected hourly, preserved at 4 °C, and necropsied within 24 h of death to determine the cause of death. The mean weight of the fish was 25 ± 5 g.

### 2.2. Bacterial Isolation and Identification

All fish were examined for specific external and internal lesions. All external lesions were examined microscopically for parasitic infections, and signs of specific bacterial infections such as *Flavobacterium* infection were examined macroscopically. To rule out certain known diseases capable of inducing mass mortality in rainbow trout, specific primers were used for testing. For viral diseases, infectious hematopoietic necrosis (IHN), infectious pancreatic necrosis (IPN), and viral haemorrhagic septicaemia (VHS) were ruled out by screening [10], and bacterial *Flavobacterium psychrophilum-*, *Pseudomonas putida-*, and *Lactococcus garviae*-related infections were ruled out using polymerase chain reaction PCR [11]. The kidneys, liver, and spleen were separated from each fish for bacterial isolation. The tissue samples were homogenized in 300 µL phosphate-buffered saline (PBS), and 100 µL was inoculated separately onto tryptic soy agar (TSA) and Cytophaga agar (both from BD Difco, Franklin Lakes, NJ, USA). The agar plates were incubated at 20 °C and 25 °C for 48 h under aerobic conditions using a Sanyo MIR-153 incubator (Sanyo, Japan). Two dominant bacteria that compromised the largest portions of the cultures were selected to be sub-cultured. Pure cultured bacterial colonies were selected for identification and were preserved at −80 °C in glycerol. In addition, to confirm the identification, bacteria glycerol stocks were grown for 18 h on TSA under the same conditions used during the original isolation, and the species were verified using MALDI Biotyper 3 (Bruker Daltonics, Fremont, CA, USA) [12].

### 2.3. 16S rRNA Gene Sequencing

To identify the bacteria, DNA was extracted from the pure cultured colonies, dissolved in 300 µL TE buffer, heated to 100 °C for 20 min, and then centrifuged at 8000× *g* for 10 min. The pellets were discarded, and the supernatants (100 µL) were analyzed by PCR using universal primers specific for the 16S rRNA sequence (24F and 1492R) [13]. For analysis of the gene sequences, the PCR products were sent to the genomic division of Macrogen (Republic of Korea) where nucleotide sequencing reactions were performed using an ABI PRISM 3730XL Analyzer and a BigDye ^®^ Terminator v3.1 cycle sequencing kit (both from Applied Biosystems, Waltham, MA, USA). The results of the sequencing were analyzed using the Basic Local Alignment Search Tool (BLAST) to identify the specific bacteria. The sequences were aligned using CLUSTALX [14] from GENEIOUS v.9.1.8 [15]. Partial sequences of the 16S rRNA gene were used to construct a phylogenetic tree, with the consensus sequences being imported into the MEGA 7.0 software. Briefly, the alignments were edited in the MEGA 7.0 software, a bootstrap analysis was performed with 1000 replications, and the phylogenetic tree was constructed using the neighbor-joining method [16].

### 2.4. Pathogenicity Challenge Trials

Rainbow trout of an average weight of 20 g were purchased from other trout farms located in Kangwon Province, Korea. The bacteria used for the experimental infections were prepared in tryptic soy broth (TSB) with incubation at 25 °C for 24 h under aerobic conditions and centrifugation at 8000× *g* for washing just prior to injection using PBS as a diluent. Titration of the bacteria was performed by optical density using a SmartSpec^TM^ 3000 spectrophotometer (Bio-Rad, Hercules, CA, USA). Groups of 10 fish were intraperitoneally injected with 3 × 10^7^, 10^6^, 10^5^, or 10^4^ colony-forming units (CFU) of bacteria suspended in 0.1 mL of PBS per fish. An equal volume of PBS was injected into each fish of the control group. All experimental infection experiments were simultaneously performed in triplicate, and the challenged fish in each group were maintained in 120 L water tanks. The temperature of the water was kept at 19 °C to 20 °C using a titanium heater, and the tanks of each group were individually aerated. The trials were conducted for 15 days, and fish showing clinical signs, such as lethargy or skin darkening, were selected for re-isolation of bacteria using the same conditions described in the bacterial isolation step.

### 2.5. Histopathological Findings

All fish were observed at intervals of 6 h every day, and those that were imbalanced or lethargic were collected immediately for examination. The lethargic fish selected during the challenge trial were prepared for histopathological examination. Briefly, the kidney, liver, and spleen were separately excised from the fish and preserved in 10% neutral-buffered formalin. Fixed tissues were trimmed, dehydrated using ethanol, and embedded in paraffin blocks, which were sectioned and stained with haematoxylin and eosin for the analysis. The specimens were examined using light microscopy and digitally scanned by Xenos Inc (Seoul, Korea).

### 2.6. Antibiotic Susceptibility Test

Bacterial strains isolated from diseased fish were subjected to antibiotic susceptibility tests. *Janthinobacterium* spp. are known to normally be highly resistant to multiple antibiotics, especially β-lactam antibiotics, which confers a pathogenic potential to *Janthinobacterium* spp. in clinical studies [17]. Strains were cultured on Mueller–Hinton agar and tested for antibiotic susceptibility using the disc-diffusion method. Each strain was individually tested against 24 antibiotics, and the results were analyzed as described in guideline M100 of the Clinical & Laboratory Standards Institute [18]. The following antibiotics were used in the study: amikacin, ampicillin, piperacillin, cefazolin, cefepime, cefotaxime, cefoxitin, ceftazidime, ceftizoxime, aztreonam, imipenem, meropenem, gentamicin, kanamycin, streptomycin, tetracycline, doxycycline, ciprofloxacin, nalidixic acid, norfloxacin, ofloxacin, trimethoprim–sulfamethoxazole, chloramphenicol, and erythromycin.

## 3. Results and Discussion

### 3.1. Identification of the Bacteria

**Figure 1:**
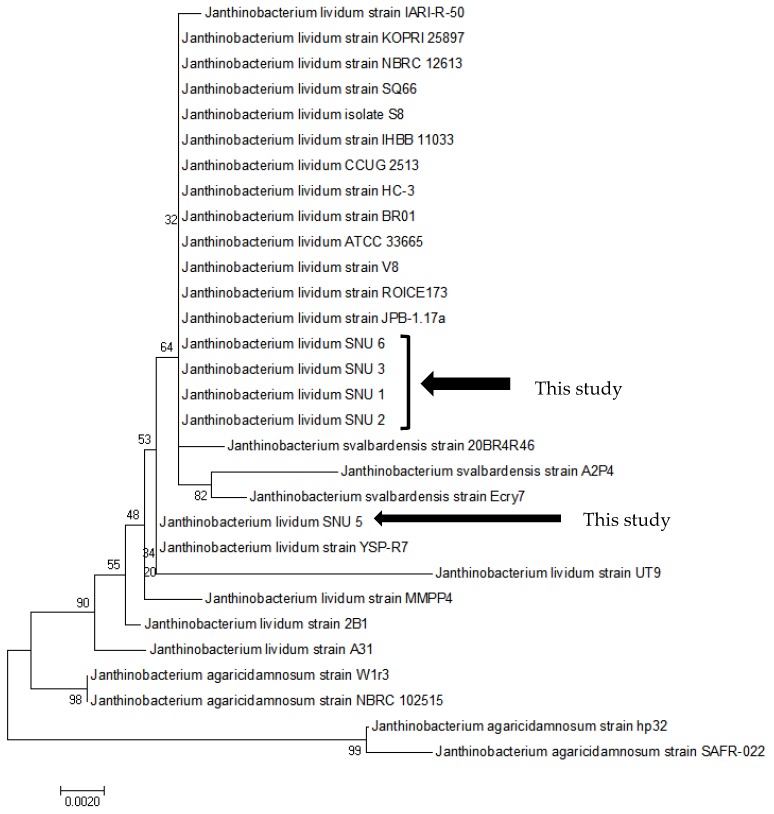
Phylogenetic tree constructed using partial 16S rRNA gene sequences (1039 bp) of *Janthinobacterium* spp. The neighbor-joining method with a bootstrap test of 1000 replicates was performed using MEGA 7.0 (GenBank accession number: MK757609, MK757610, MK757611, MK757613, MK757614).

The phylogenetic tree constructed using sequence information from the cultured isolates identified the etiological bacterium as *J. lividum* (Figure 1). The results were confirmed using matrix-assisted laser desorption/ionization (MALDI) Biotyper 3 software version 3.1 (Bruker, Fremont, CA, USA). The 16S rRNA gene of the isolates had 99.93% similarity to the *J. lividum* DSM 1522 type strain [19].

### 3.2. Pathogenicity Challenge Trials and Clinical Signs

**Figure 2:**
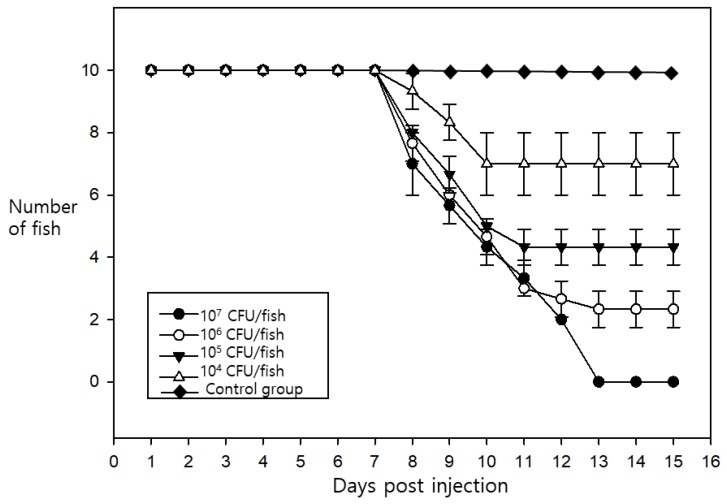
Pathogenicity during the challenge trials. The fish were inoculated intra-peritoneally. The inoculating dose of bacteria was administered to 4 groups of 10 fish/group (3 × 10^7^ colony-forming units (CFU)/fish, 10^6^ CFU/fish, 10^5^ CFU/fish, and 10^4^ CFU/fish). The trial was performed in triplicate. The results shown are the average survival rates for the indicated groups.

In pathogenicity challenge trials, the estimated half-maximal lethal dose (LD_50_) was 3 × 10^5^ CFU/fish (Figure 2), which was higher compared to 10^2^ CFU/fish observed in a previous study [7]. The overall survival of the fish was 0% when the titer was greater than 3 × 10^7^ CFU/fish. The death of the fish started to occur at 7 days post-infection, regardless of the dose of the injected bacteria. However, no specific external or internal lesions were observed during the test. The behavioral and physical traits of the infected fish were clearly abnormal, including a loss of appetite, lethargic swimming movements, and darkening of the skin. General clinical signs presenting in the diseased fish included spleen enlargement; however, specific signs such as haemorrhaging or ascites fluid were not observed.

### 3.3. Histopathologic Analysis

**Figure 3:**
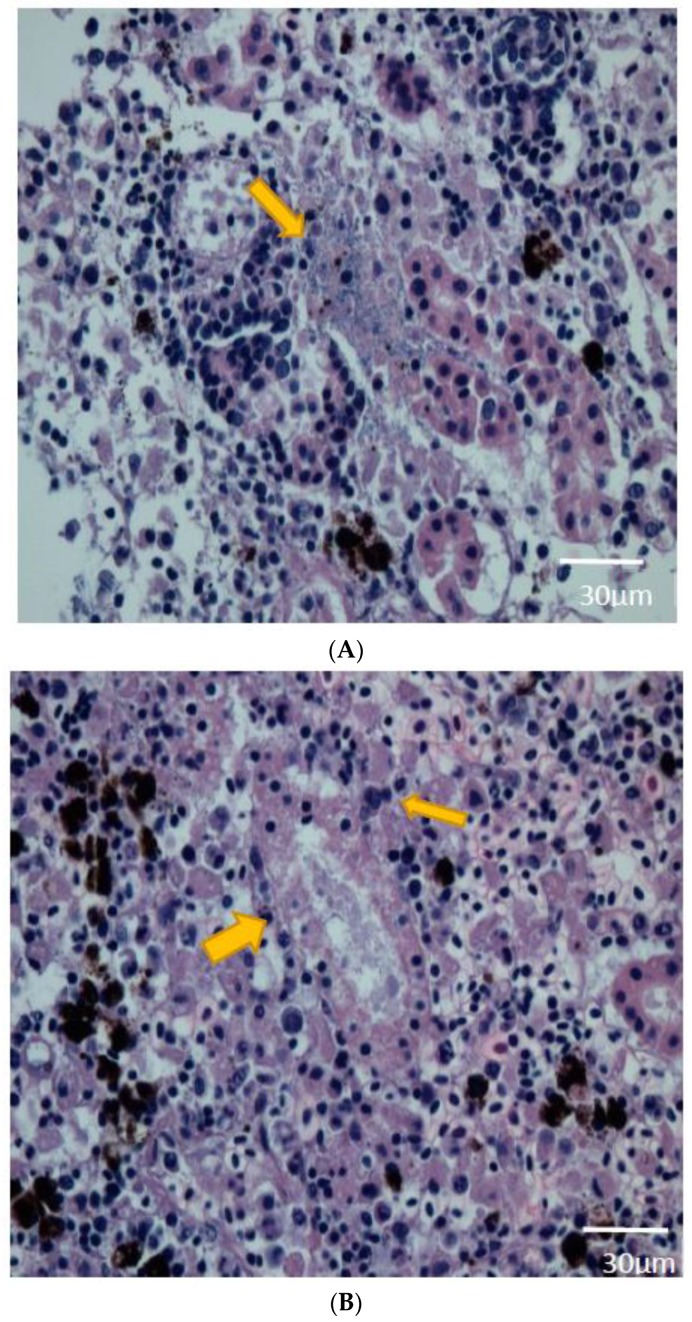
(**A**) Bacteraemia in the interstitial area of the kidney. (**B**) Tubular degeneration and necrosis caused by infection with *Janthinobacterium lividum*, with infiltration of lymphocytes and macrophages. (haematoxylin and eosin staining).

During histopathologic examination of kidney tissue from the experimentally infected fish, definite signs of tubular degeneration were observed, and bacteria were present near the tubules in every fish. In addition, there was an increase in the number of immunocytes, such as melanomacrophages, lymphocytes, and neutrophils. The infiltrating cells typically were observed along the tubule structure of the kidney (Figure 3A,B). None of the histopathologic signs noted in the kidneys were observed in the liver or spleen of the same individuals.

For the identification of bacteria that infiltrated the tubule structure, the bacteria were re-isolated with the same procedure used for their initial isolation from the kidney and liver tissues of euthanized lethargic fish. The bacteria recovered from the re-isolation were confirmed to be *J. lividum*. To verify the pathogenicity of the bacteria in the experimental fish, the same procedure was performed in the mock-infected control group. The histopathologic results were clear, and no bacteria were isolated from any of the control group fish.

### 3.4. Antibiotic Susceptibility

Antibiotic susceptibility tests provide important information for the prevention and proper treatment of infections. Results from previous studies [4] indicate that *J. lividum* is resistant to multiple antibiotics but it is susceptible to doxycycline and trimethoprim–sulfamethoxazole. Furthermore, *J. lividum* shows the highest level of susceptibility to trimethoprim–sulfamethoxazole. To compare one of the current isolates (strain SNU 1) with the ATCC 12,473 strain, the antibiotic disk-diffusion test was performed under the same conditions. The results demonstrated that the strain SNU 1 was resistant to multiple antibiotics, including cefepime, cefotaxime, gentamicin, amikacin, streptomycin, tetracycline, ciprofloxacin, nalidixic acid, norfloxacin, ofloxacin, and chloramphenicol (Table 1).

Various species of bacteria have been isolated from samples of river and lake water [20]. Some of the isolates that are considered part of the normal flora of the environment, such as *Aeromonas* species including *Aeromonas hydrophila* and *Aeromonas salmonicida*, can be fatal pathogens for aquatic animals such as salmon species [20]. *J. lividum* also commonly exists in aquatic environments and can be part of the normal gut microbiota of rainbow trout [8]. However, *J. lividum* can also be an opportunistic pathogen of rainbow trout under unbalanced conditions caused by stress or wounds to the fish. Surprisingly, only one previous study has investigated the pathogenicity of *J. lividum* [7], even though its other characteristics, such as its effective inhibitory actions and its other uses in various fields, have been extensively studied.

*J. lividum* is normally considered to be non-pathogenic to amphibians, which may be one of the main reasons why it has been used for treating fungal diseases in the amphibian-rearing industry. However, the potential for *J. lividum* to be an opportunistic pathogen of rainbow trout should not be ignored within the fish industry, especially if this bacterium is capable of inducing bacteraemia in the kidney of fish. Previous studies have indicated that the LD_50_ of these bacteria is approximately 10^2^ CFU/fish and the main clinical sign of the infection is septicaemia [7]. In contrast, the LD_50_ in our current study was higher, and none of the experimentally inoculated fish presented with signs of septicaemia or died on the first day of infection.

According to reports from the Food and Agriculture Organization (FAO) of the United Nations (http://www.fao.org/fishery/culturedspecies/Oncorhynchus_mykiss/en), the global consumption of rainbow trout is continually increasing also in Asian countries such as Japan, China, India, and Taiwan. This has resulted in a steady growth of the rainbow trout industry in Korea. Although extensive research regarding the rainbow trout is being conducted in many different countries in Asia, there have been no previous reports concerning *Janthinobacterium* species as a pathogen in trout fisheries, which may be a result of the bacteria’s specific location and distribution. It is important to note that since *Janthinobacterium* species have been previously reported as pathogens in trout fisheries in European countries, its emergence in Asian rainbow trout fisheries, imported from other countries, is possible. In addition, *Janthinobacterium* has been reported as a hospital-acquired pathogen that can cause septicemia in patients [17]. Due to the fact that some Asian countries, including Korea, consume rainbow trout raw, its potential effect on human health should not be neglected.

The antibiotic susceptibility test showed that the strain isolated in the current study was much more resistant to antibiotics than the CCUG 2344 strain, which indicates that the overuse of antibiotics may have contributed to the current situation in Korean rainbow trout fisheries. Based on our findings, we conclude that the pathogenicity and infection process of *J. lividum* may vary depending on the specific properties of each strain. Further research on the pathogenic functions of these bacteria is warranted, as well as investigations into potential treatments and prevention strategies using other antibiotics. The findings from these studies could create considerable profits for rainbow trout fisheries that may be experiencing damage and losses caused by *J. lividum*.

## 4. Conclusions

To conclude, our study focused on the pathogenic role and histologic analysis of *J. lividum* infection in rainbow trout. Because not many studies have been conducted on the pathogenicity of the bacterium, the potential possibility of its role as an opportunistic pathogen has been overlooked. Also, this is first study reporting *J. lividum* as a causative agent of mortality of rainbow trout in farms located in Korea. Furthermore, since there is no additional report indicating infections of *J. lividum* in Asia countries, it is possible that the disease spreads sporadically in Asia. The pathogenicity of the bacteria was proved using challenge trials and histopathological analyses. Also, the antibiotic susceptibility test results on the isolated strain indicated that the bacterium was gaining resistance to multiple antibiotics. Further study considering the epidemiological distribution and pathogenic role in other fish species seems to be needed for the prevention and control of disease resulting from *J. lividum* infection.

## Figures and Tables

**Table 1:** Antibiotic susceptibility test results for *J. lividum* strain SNU 1 by the disk-diffusion method.

Antibiotics	AMP	PIP	CFZ	FEP	CTX	FOX	CAZ	ZOX
Resistance	R	R	R	R	R	R	R	R
Inhibition zone diameter (mm)	10	11	9	5	10	15	13	13
Antibiotics	ATM	IPM	MEM	GEN	AMK	KAN	STR	TET
Resistance	R	R	R	R	R	R	R	I
Inhibition zone diameter (mm)	9	10	11	6	8	8	7	14
Antibiotics	DOX	CIP	NAL	NOR	OFX	TMP	CHL	ERY
Resistance	S	R	R	R	R	S	R	R
Inhibition zone diameter (mm)	17	13	5	8	9	20	6	11

## Abbreviations

AMP	ampicillin
PIP	piperacillin
CFZ	cefazolin
FEP	cefepime
CTX	cefotaxime
FOX	cefoxitin
CAZ	ceftazidime
ZOX	ceftizoxime
ATM	aztreonam
IPM	imipenem
MEM	meropenem
GEN	gentamicin
AMK	amikacin
KAN	kanamycin
STR	streptomycin
TET	tetracycline
DOX	doxycycline
CIP	ciprofloxacin
NAL	nalidixic acid
NOR	norfloxacin
OFX	ofloxacin
TMP	trimethoprim sulfamethoxazole
CHL	chloramphenicol
ERY	erythromycin
R	resistant
I	intermediate
S	susceptible
